# Hand reach star excursion balance test: An alternative test for dynamic postural control and functional mobility

**DOI:** 10.1371/journal.pone.0196813

**Published:** 2018-05-08

**Authors:** Ola Eriksrud, Peter Federolf, Patrick Anderson, Jan Cabri

**Affiliations:** 1 Department of Physical Performance, Norwegian School of Sports of Science, Oslo, Norway; 2 Department of Sport Science, University of Innsbruck, Innsbruck, Austria; University of Salzburg, AUSTRIA

## Abstract

Tests of dynamic postural control eliciting full-body three-dimensional joint movements in a systematic manner are scarce. The well-established star excursion balance test (SEBT) elicits primarily three-dimensional lower extremity joint movements with minimal trunk and no upper extremity joint movements. In response to these shortcomings we created the hand reach star excursion balance test (HSEBT) based on the SEBT reach directions. The aims of the current study were to 1) compare HSEBT and SEBT measurements, 2) compare joint movements elicited by the HSEBT to both SEBT joint movements and normative range of motion values published in the literature. Ten SEBT and HSEBT reaches for each foot were obtained while capturing full-body kinematics in twenty recreationally active healthy male subjects. HSEBT and SEBT areas and composite scores (sum of reaches) for total, anterior and posterior subsections and individual reaches were correlated. Total reach score comparisons showed fair to moderate correlations (r = .393 to .606), while anterior and posterior subsections comparisons had fair to good correlations (r = .269 to .823). Individual reach comparisons had no to good correlations (r = -.182 to .822) where lateral and posterior reaches demonstrated the lowest correlations (r = -.182 to .510). The HSEBT elicited more and significantly greater joint movements than the SEBT, except for hip external rotation, knee extension and plantarflexion. Comparisons to normative range of motion values showed that 3 of 18 for the SEBT and 8 of 22 joint movements for the HSEBT were within normative values. The findings suggest that the HSEBT can be used for the assessment of dynamic postural control and is particularly suitable for examining full-body functional mobility.

## Introduction

Different tests of dynamic postural control have gained popularity and interest since they are considered more ecological in sports or physical activities [1]. One such test is the star excursion balance test (SEBT) which was originally presented as a low-cost rehabilitation tool [2]. The SEBT quantifies maximum foot reach distances of the non-stance foot using a star on the ground with 8 different reaching directions at 45-degree intervals extending from a center point [3]. Currently, the star excursion balance test (SEBT) is a well-established task-based objective clinical test battery of dynamic postural control that measures different aspects of neuromuscular functions, such as proprioception [4], strength [5–7], power [8], balance [6] and coordination [9] while eliciting different combinations of trunk and lower extremity joint movements [10–14]. Clinical application of the SEBT has primarily focused on lower extremity joint dysfunctions such as ankle instability, knee dysfunction after anterior cruciate ligament reconstruction, patella femoral pain and in the prediction lower extremity injuries [1]. The SEBT is frequently described as a “series of single leg squats” [1], and is therefore not well suited to capture movements in the transverse plane, as is reflected by elicited hip rotational joint movements [10, 12, 15]. Furthermore, SEBT neither captures all hip joint movements nor does it represent the interaction of larger trunk and upper extremity joint movements.

Complementing the SEBT with hand reaches is a justifiable approach to reduce these shortcomings. However, current hand reach tests also have shortcomings since they are performed in bilateral stance and elicit neither large joint movements nor vertical displacement of the center of mass (COM) [16–18]. Hand reaches based on SEBT reaching directions, the “hand reach star excursion balance test” HSEBT [19], may provide a platform in which upper extremity and greater trunk movements are integrated with lower extremity joint movements. Consequently, the HSEBT can complement the clinical application of the SEBT by addressing full body movements in the assessment of dynamic postural control. In addition, these hand reach tests can also serve as a measure of functional mobility, i.e. the combination of range of motion (ROM) of multiple joints utilized to accomplish more ecological activities of daily living and athletic performance. If HSEBT reaches are to be a measure of functional mobility they should elicit more and greater joint movements than their SEBT counterparts. Also, the elicited joint movements from the HSEBT should be more comparable to established normative ROM goniometric reference data, indicating that mobility is being challenged. Thus, information obtained from HSEBT reaches can provide clinicians with a systematic assessment tool to better understand the influence of dysfunction such as shoulder instability [20] and low back pain (LBP) [21] on full body movements.

The purpose of the current study was to 1) determine if the HSEBT reaches provide different information about dynamic postural control than the SEBT reaches, and 2) compare joint movements elicited by HSEBT to both SEBT and normative joint mobility (ROM) values published in the literature.

## Materials and methods

### Participants

A convenience sample of twenty recreationally active healthy male subjects (age 24.4 ± 2.3 years; height 179.9 ± 6.0 cm; weight 77.5 ± 9.3 kg; mean ± SD) volunteered for the study. Exclusion criteria were musculoskeletal or neurological dysfunction or injury in the past six months. Body height and weight were obtained using a Seca model 217 stadiometer and a Seca flat scale (Seca GmbH. & Co. Hamburg. Germany).

#### Ethics approval

The committee for medical and health research ethics in Norway (2012/1736) and Norwegian Data Protection Agency (40996) approved the study. Measurements were carried out according to the principles described in the Declaration of Helsinki. All subjects were given written and verbal information about the study prior to giving written informed consent. The individual in this manuscript has given written informed consent (as outlined in PLOS consent from) to publish these case details.

### Experimental design

Descriptive and cross-sectional cohort study for comparison of HSEBT and SEBT reach tests.

### Procedures

The HSEBT consists of 10 hand reaches on each foot (stance foot) with toe-touch of the opposite foot in the same 8 directions as used for the SEBT with the addition of two rotational reaches. HSEBT reaching directions are defined from the anatomical neutral position as follows: direction (i.e.: anterior (A); posterior (P)), side of body (left (L); right (R)), angle at 45° increments from anterior (0°) to posterior (180°) and movement (rotation (ROT)). Reaches along the 8 horizontal reach vectors (A0, R45, R90, R135, P180, L135, L90 and L45) are horizontal reaches (HR) and measured in centimeters (cm), while the two rotational reaches (LROT, RROT) are measured in degrees (°). These reach definitions were applied to the SEBT for ease of comparison, which differs from established SEBT definitions based on stance foot [3]. Furthermore, two rotational reaches were added to the SEBT to complement the HSEBT rotational reaches, and to target transverse plane dynamic postural control in single leg stance. Both HSEBT and SEBT reaches can be classified based on plane(s) of motion: pure plane (A0, P180, L90, R90, LROT, RROT) and diagonal (L45, R45, L135, R135); or with subgroups based on direction of movement: anterior (L45, A0, R45), posterior (L135, P180, R135), lateral (L90, R90), and rotational (LROT, RROT).

HSEBT and SEBT reaches were performed in the same order and executed on a testing mat, which was developed to guide and measure the different reaches. The mat was imprinted with horizontal reaching directions marked at 2 cm intervals and with nine concentric circles at 10 cm intervals marked at 5-degree intervals (Athletic Knowledge Nordic AB, Stockholm, Sweden). Both the HSEBT and SEBT testing procedures are described in detail elsewhere [3, 19]. The following clarifications concerning the SEBT need to be made:1) the stance foot was placed on the middle of the mat, 2) heel, first and fifth metatarsal heads maintained ground contact during the reaches, 3) the trunk aligned with the reach vector for diagonal reaches (R45, R135, L135 and L45); 4) the lateral reaches (R foot L90 and L foot R90 reach) were performed with the reaching foot in front of stance foot, and additionally 5) during rotational reach the big toe of the reaching foot followed the 50 cm radius circle with its longitudinal axis oriented toward the center of the testing mat. For all HSEBT and SEBT reaches a minimum of three practice trials were allowed, after which three valid maximum reaches were recorded of which the highest value was used for analysis. Trials were discarded if the procedures were not followed.

**Fig 1:**
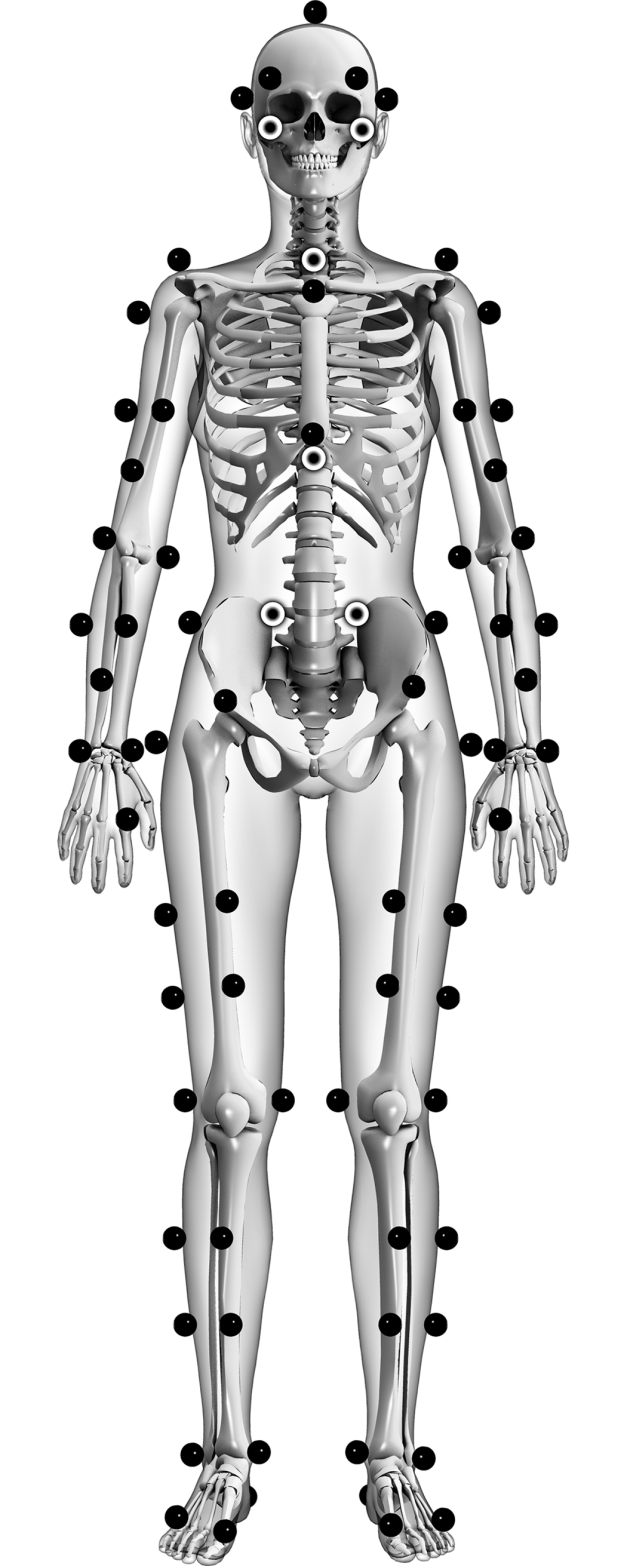
Marker set used for kinematic data acquisition.

Kinematic data of all reaches were obtained using 15 Oqus cameras (ProReflex®, Qualisys Inc., Gothenburg, Sweden) recording at 480 Hz. Fifty-eight spherical reflective markers (20 mm Ø) were attached over specific anatomical landmarks (Fig 1) to define and track the foot, leg, thigh, pelvis, thorax and upper arm segments. The marker clusters used for the leg, thigh and upper arm were attached firmly using tensoplast elastic tape (BSN Medical GmBH, Hamburg, Germany). The markers were identified using the Qualisys software (Qualisys Inc., Gothenburg, Sweden). To minimize the risk of gaps in marker trajectories, especially for the anterior trunk and pelvic markers during anterior reaches (L45, A0 and R45), lateral pelvic markers were included in the marker set for tracking and four Qualisys cameras were placed as close to the ground as possible. If gaps in marker trajectories occurred, they were interpolated or reconstructed [22]. However, these methods sometimes failed with a minimum number of subjects included for HSEBT shoulder (14), trunk (19), hip (20), knee (19) and foot (20) joint movement calculations. All joint movement calculations for the SEBT included all subjects, except for LROT (19) and RROT (18). Otherwise, the marker data were not treated or filtered.

### Data analysis

The data analysis was carried out using Visual 3D® (C-Motion Inc., Rockville, MD, USA). Local coordinate systems for the foot, leg, thigh, pelvis, thorax and upper arm were created [23, 24]. Three-dimensional joint rotations of the ankle, knee, hip and trunk were then calculated (cardan sequence XYZ). Shoulder motions were calculated using both ZYZ (Z_first_ = horizontal adduction and abduction, Y = abduction and adduction, Z_third_ = internal and external rotation) and XYZ (X = flexion and extension) cardan sequences. Prior to reaching the subjects were asked to stand feet parallel to shoulder line with hands on the hips for a minimum of 3 seconds. Normalization of joint starting positions was defined as the mean joint positions observed during the last 95 of the first 100 frames of recording (ϕ_start_) (Eq 1).

$$\phi_{start}={mean}_{frames\;5-100}$$ (1)

The local coordinate system of the upper arm was aligned with the thorax at the beginning of each motion trial, and was used for all joint angle calculations of the shoulder. Furthermore, the neutral starting position for shoulder horizontal abduction and adduction was defined as the upper arm oriented in the frontal plane (90° abducted position). Maximum reach position (ϕ_max_) was defined as the highest (or lowest) x, y and z-coordinate values in the global coordinate system of the second metacarpal and the first metatarsal marker of the reaching hand or foot, respectively, with procedures described in detail elsewhere [19]. All tests were visually inspected to ensure that the set criteria matched for ϕ_max_. Joint movements (θ) were then calculated (Eq 2) for each reach and averaged for all subjects.

$$\theta =\phi_{max}-\phi_{start}$$ (2)

Joint movements of mirrored reaches (left and right) were averaged and named based on left stance foot definitions for ease of data presentation. In tests with bilateral symmetrical shoulder joint movements, i.e. A0, P180, L90 and R90 reaches, only the mean of left and right shoulders is presented. Reaches eliciting the greatest values in joint movements (θ_max_) of the ankle foot complex, knee, hip, trunk and shoulder were identified for both the HSEBT (θ_maxHSEBT_) and SEBT (θ_maxSEBT_) and their differences were calculated (θ_maxDIFF_) (Eq 3).

$$\theta_{maxDIFF}=\theta_{maxHSEBT}-\theta_{maxSEBT}$$ (3)

Then, θ_maxHSEBT_ and θ_maxSEBT_ values were compared to determine if they were within a 95% confidence interval of normative ROM reference [25], except knee rotations and trunk movements (lumbar and thoracic spine values added) were compared to the lowest reported values [26]. Comparisons of θ_maxHSEBT_ and θ_maxSEBT_ ankle and knee abduction and adduction were not done since these measures are not commonly quantified using clinically available assessment tools and normative clinical ROM values are lacking [25]. Shoulder θ_maxHSEBT_ comparisons to normative values were done for flexion, abduction, external rotation [25] and horizontal adduction [26] only. Thus, eighteen joint movements (ankle, knee, hip and trunk) were compared for both HSEBT and SEBT, with the addition of four shoulder joint movements for the HSEBT only.

Our clinical experience indicated that expressing test outcomes as areas provides a better feedback of results than composite scores. Therefore, both areas and composite scores were used in the analysis. Total area (A_tot_) was calculated as the sum of the areas covered by the 8 triangles obtained in the horizontal reach measurements (HR_i_ (i = 1(A0), 2(R45), 3(R90), 4(R135), 5(P180), 6(L135), 7(L90) and 8(L45)) (Eq 4). Additionally, anterior (A_ant_) (Eq 5) and posterior areas (A_post_) (Eq 6) were calculated in order to differentiate between anterior and posterior HSEBT reaches, respectively. Composite scores (CS) were also calculated since they have been used to quantify combinations of SEBT reaches [27]. Specifically, CS were calculated as the sum of all (CS_tot_), anterior (CS_ant_), and posterior reaches (CS_post_) (Eqs 7–9).

$$A_{tot}=\Sigma \frac{1}{2}∙{HR}_{1-8}∙{HR}_{1-8}∙{sin45}^{\circ }$$ (4)

$$A_{ant}=\Sigma \frac{1}{2}∙{HR}_{1-3,7-8}∙{HR}_{1-3,7-8}∙{sin45}^{\circ }$$ (5)

$$A_{post}=\Sigma \frac{1}{2}∙{HR}_{3-7}∙{HR}_{3-7}∙{sin45}^{\circ }$$ (6)

$${CS}_{tot}=\Sigma {HR}_{1-8}$$ (7)

$${CS}_{ant}=\Sigma {HR}_{1,2,8}$$ (8)

$${CS}_{post}=\Sigma {HR}_{4-6}$$ (9)

In order to determine similarities of movement strategies between direction specific HSEBT and SEBT reaches, shared movement synergies were quantified as the number of common joint movements (maximum 12) and defined as: strong (>8), moderate (5 to 8) and weak (<5).

Descriptive statistics (mean and standard deviation (SD)) were calculated in Excel for Mac OS 10.10.5 (Apple Inc., Cupertino, CA, USA), version 14.4.8 (Microsoft Corp., Redmond, WA, USA). All other statistical tests were done using IBM SPSS version 21.0 (IBM, Armonk, NY, USA). Normality of the data was assessed using Shapiro Wilk´s test (p<0.05). Outliers were determined and removed from the analysis [28]. The relationship between HSEBT and SEBT areas, composite score and were estimated using linear regression analysis. Interpretation of correlation coefficients was done according to the guidelines of Portney and Watkins [29]. To determine whether the differences between θ_maxHSEBT_ and θ_maxSEBT_ were different, two-sided paired t-tests (level of confidence α>95%) were used. Effect size was calculated using Cohen´s *d* (<0.2 = small; 0.2 to 0.5 = medium; >0.8 = large effect).

## Results

**Fig 2:**
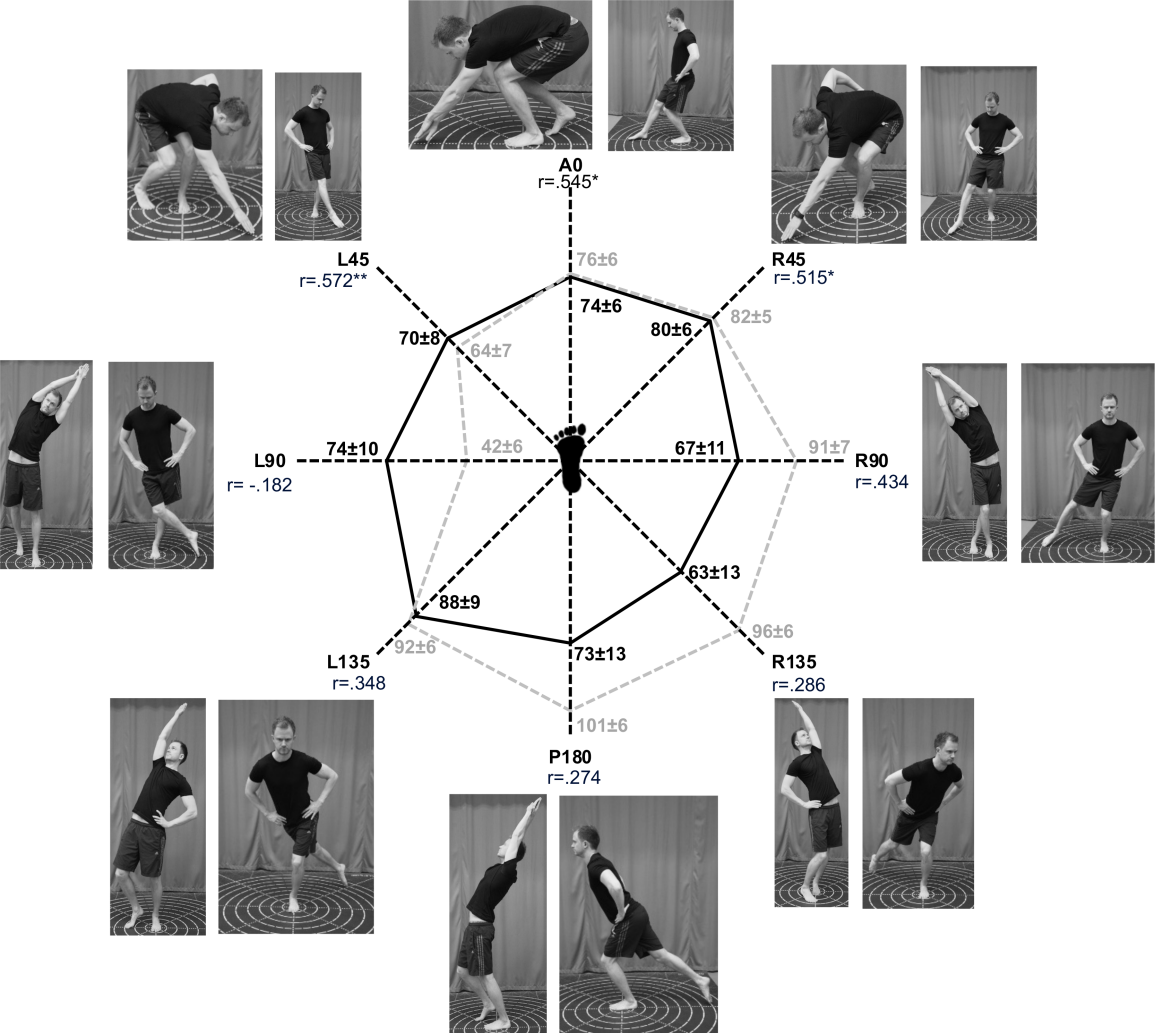
Horizontal reaches HSEBT and SEBT left leg. Visual representations of the execution of the horizontal reaches (photographs) and mean (±SD) reach distances (cm) observed for all tests in the center graphs for HSEBT (black) and SEBT (grey). Correlation coefficients (r) are shown for each direction (*p<0.05 and **p<0.01).

**Fig 3:**
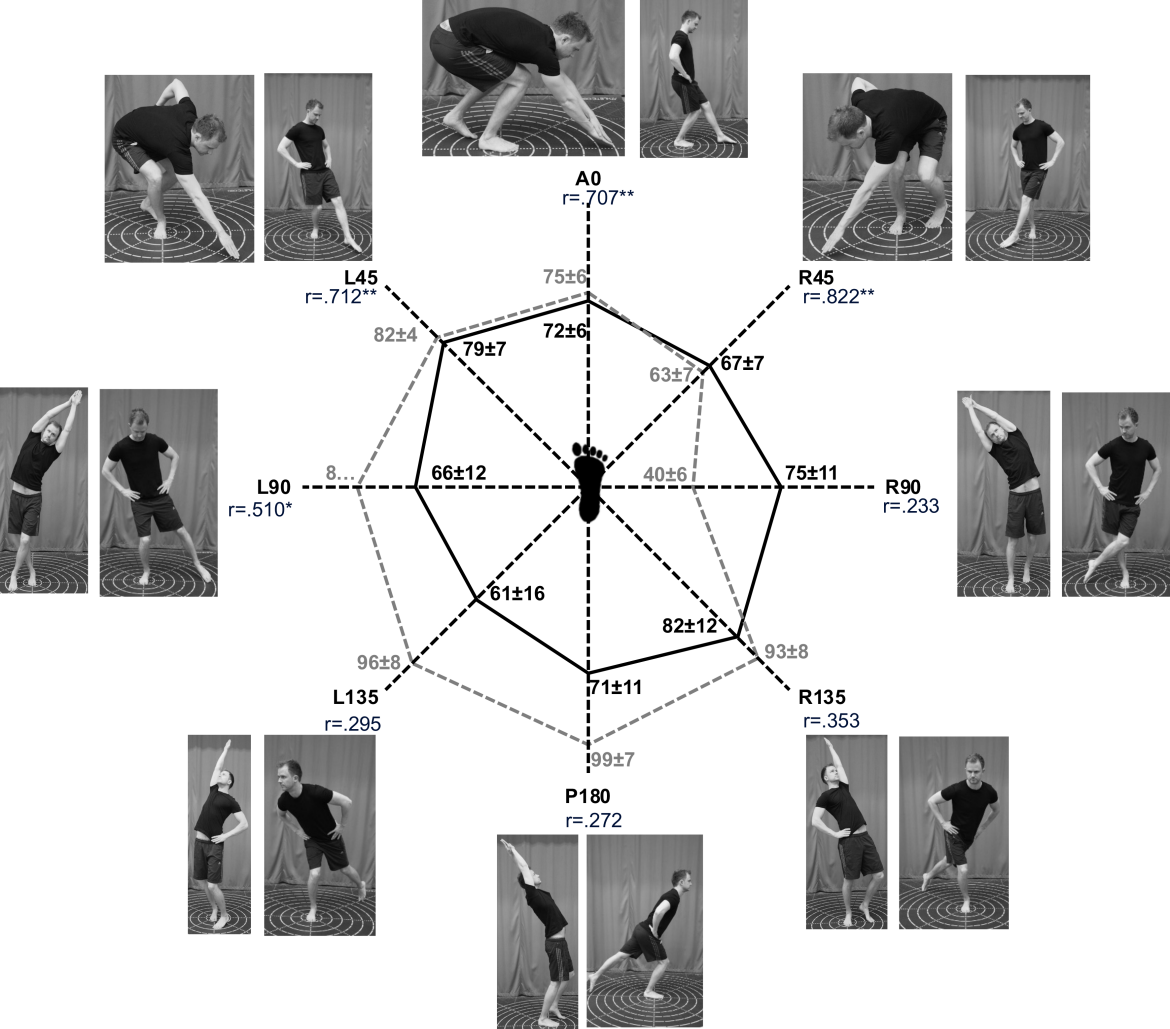
Horizontal reaches HSEBT and SEBT right leg. Visual representations of the execution of the horizontal reaches (photographs) and mean (±SD) reach distances (cm) observed for all tests in the center graphs for HSEBT (black) and SEBT (grey). Correlation coefficients (r) are shown for each direction (*p<0.05 and **p<0.01).

**Fig 4:**
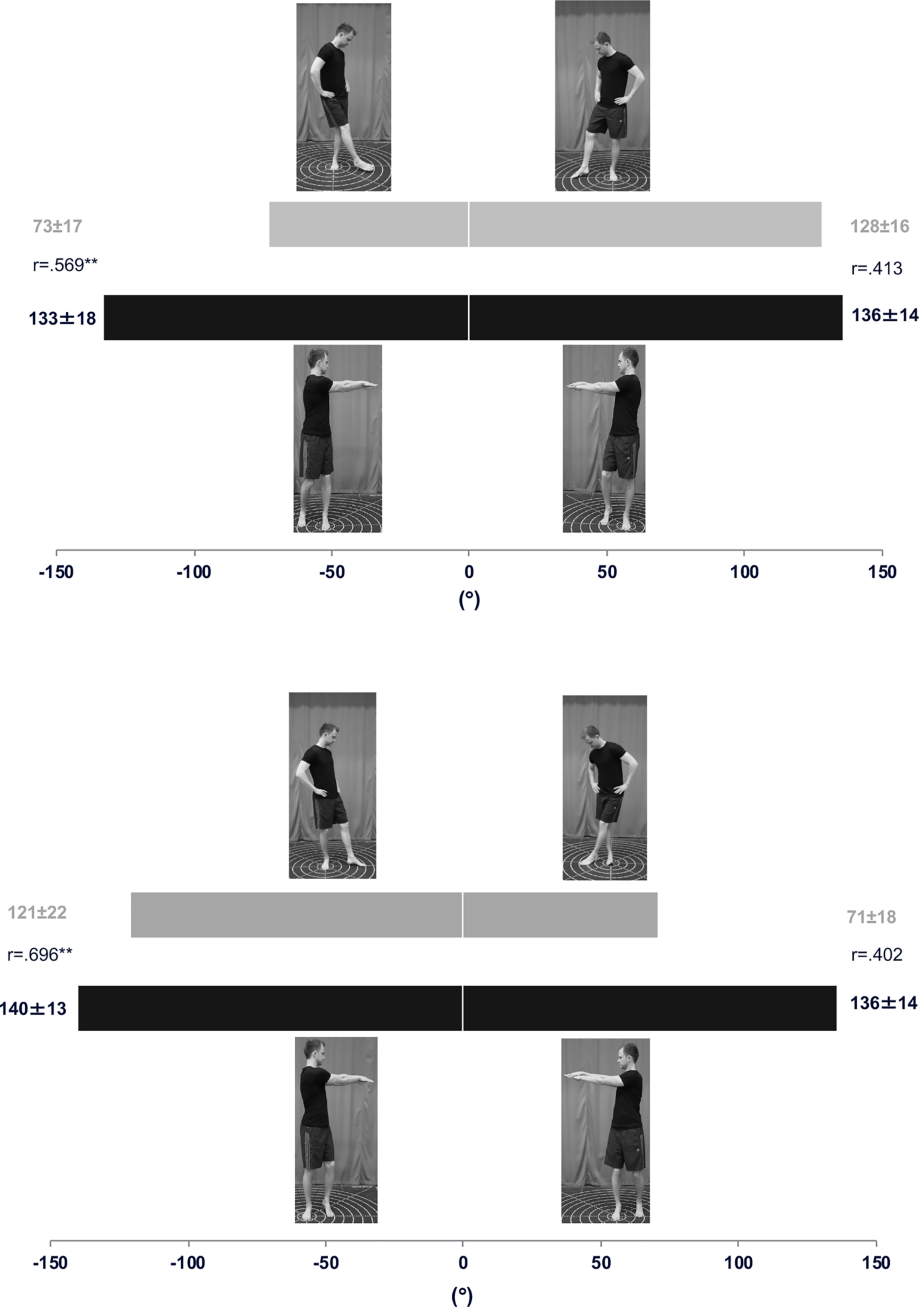
Rotational reaches HSEBT and SEBT. Visual representation of the execution of the rotational reaches (photographs) for both left (top) and right leg (bottom) with mean (± SD) reach excursion (°) observed for all tests in the horizontal bar graphs for both HSEBT (black) and SEBT (grey). Correlation coefficients (r) are shown for each direction (*p<0.05 and **p<0.01).

Test results for all HSEBT and SEBT reaches are presented in Figs 2, 3 and 4.

**Fig 5:**
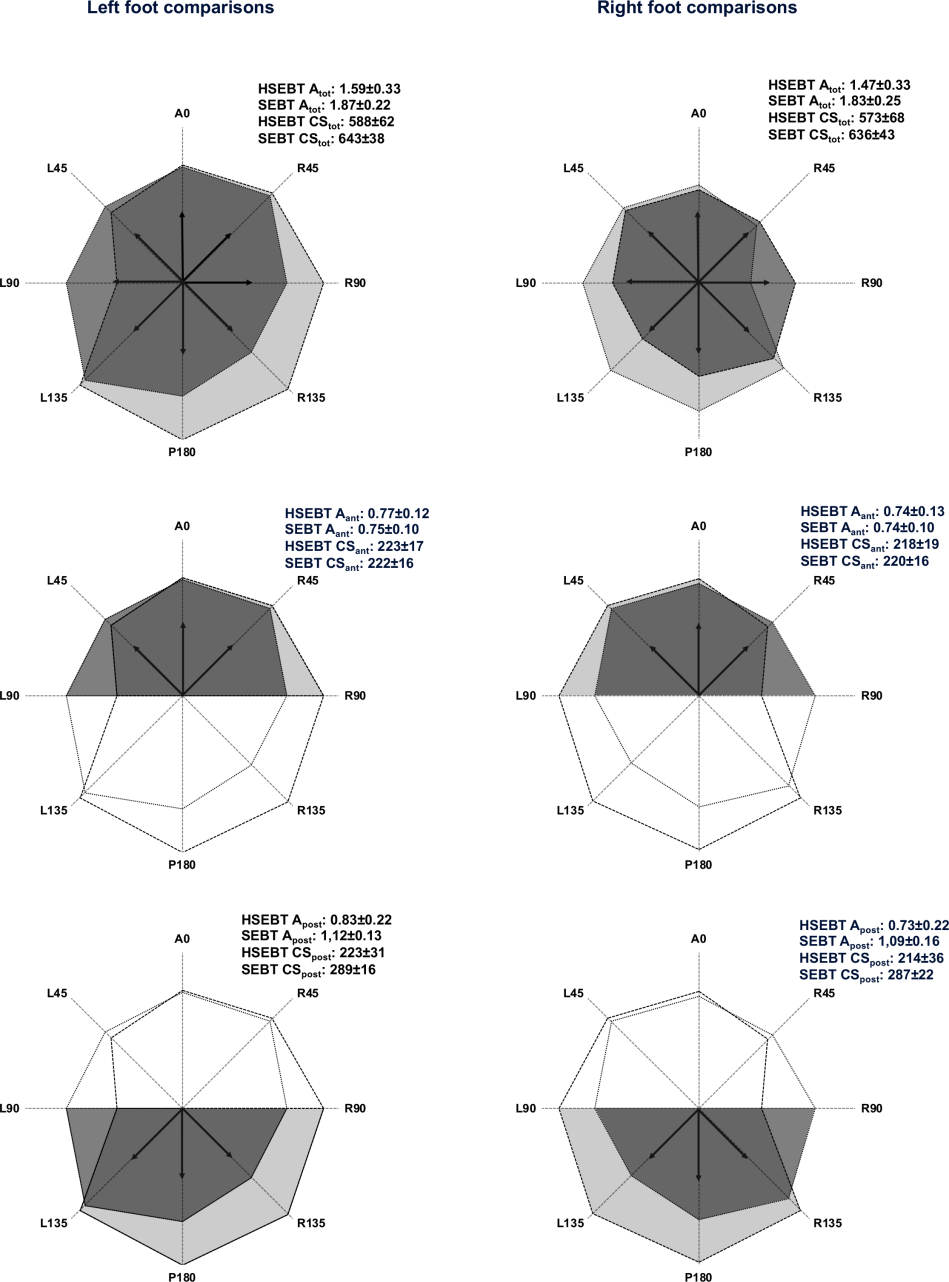
Area and composite score comparisons of HSEBT and SEBT. Visual representation of total (top row), anterior (middle row) and posterior (bottom row) comparisons of area (A_tot_, A_ant_, A_post_) and composite score (CS_tot_, CS_ant_, CS_post_). Color coding of area in the center graphs is defined as follows: dark (shared area HSEBT and SEBT), medium (unique HSEBT area) and light grey (unique SEBT area). Arrows represent the horizontal reaches included in CS_tot_.

Total area (A_tot_) and composite score (CS_tot_) correlations ranged from .393 to .606, with statistical significance for the right foot only (Table 1). Both A_ant_ and CS_ant_ have higher correlations (.531 to .823) than A_post_ and CS_post_ (.269 to .406) (Table 1 and Fig 5). Anterior reaches, both on the left and right foot, had moderate to good correlations ranging from r = .515 to .572 and r = .707 and .822, respectively. None of the posterior reaches were significantly correlated (Figs 2 and 3). Anterior hand reach to posterior foot reach (A and CS) was significantly correlated (.534 to .698), while posterior hand reaches to anterior foot reaches (A and CS) was significantly correlated for the right foot only (.469 and .480) (Table 1). Variable correlations were observed for the lateral (-.182 to .510) and rotational reaches (.402 to .696).

**Table 1:** Area and composite score comparisons between HSEBT and SEBT.

	Left foot		Right foot	
Comparisons	r	R^2^	r	R^2^
A_tot_	.393.	.154	.602**	.362
A_ant_	.531*	.282	.780**	.608
A_post_	.269	.072	.406	.165
HSEBT A_ant_ and SEBT A_post_	.534*	.285	.698**	.487
HSEBT A_post_ and SEBT A_ant_	.227	.052	.480*	.230
CS_tot_	.414	.171	.606**	.367
CS_ant_	.605**	.366	.823**	.677
CS_post_	.341	.116	.344	.118
HSEBT CS_ant_ and SEBT CS_post_	.536*	.287	.608**	.370
HSEBT CS_post_ and SEBT CS_ant_	.261	.068	.469*	.220

A_tot_, Total area; A_ant_, Anterior area; A_post_, Posterior area; CS_tot_, Total composite score; CS_ant_, Anterior composite score; CS_post_, Posterior composite score.

* p<0.05

** p<0.01

A detailed description of elicited joint movements of both the HSEBT and the SEBT with reach specific comparisons is presented in Table 2. HSEBT anterior reaches resulted in ankle dorsiflexion (19.4–29.7°), knee flexion (81.6–101.7°), hip flexion (98.8–103.3°) and trunk flexion (51.2–58.8°), while posterior reaches elicited ankle dorsiflexion (19.7–24.5°), knee flexion (18.0–28.8°), hip extension (17.4–29.5°) and trunk extension (28.5–36.2°). HSEBT lateral reaches targeted different frontal plane movements where the L90 reach generated ankle inversion (7.5±4.5°), knee abduction (2.1±3.7°), hip abduction (16.9±6.3°) and ipsilateral trunk flexion (38.2±7.0°), whereas the R90 reach elicited ankle eversion (18.2±3.3°), knee adduction (2.7±3.0°), hip adduction (27.6±6.4°) and contralateral trunk flexion (38.8±5.8°). HSEBT rotational reaches targeted different transverse plane movements where the LROT reach induced ankle adduction (15.1±5.2°), knee internal rotation (15.1±3.7°), hip internal rotation (33.2±3.8°), whereas the RROT reach elicited ankle abduction (13.4±3.6°), knee external rotation (23.8±5.4°), hip external rotation (29.5±5.4°) and contralateral trunk rotation (33.7±4.5°). Shoulder extension, adduction, internal rotation and horizontal abduction are not reported since no test targeted these movements specifically and the observed θ_maxHSEBT_ were small.

**Table 2:** Kinematic comparisons HSEBT and SEBT.

Test	Direction	Plane of motion	Ankle θ (°)	Knee θ (°)	Hip θ (°)	Trunk θ (°)	Shoulder θ (°)	Movement synergy
HSEBT	A0	Sag	**DF:26.2±4.5**	**Flex:101.7±16.0**^**a**^	**Flex: 103.3±19.8**	**Flex: 58.8±9.7**^**a**^	Flex: 112.9±11.3	8/12
		Front	**Ev:12.9±5.8**	**Add: 13.2±7.8**	Abd: 1.0±7.4	Ipsi lat flex: 4.1±4.5		
		Trans	**Abd:6.1±3.2**	**IR: 12.9±10.4**	ER: 1.7±5.5	Ipsi rot: 0.9±3.6		
SEBT	A0	Sag	**DF: 31.0±4.7**	**Flex: 64.9±11.2**	**Flex: 24.4±16.0**	**Flex: 3.5±16.5**		
		Front	**Ev: 4.3±3.2**	**Add: 3.8±6.4**	Add: 16.6±5.1	Contra lat flex: 2.0±10.4		
		Trans	**Abd: 7.4±2.0**	**IR: 3.2±4.5**	IR: 11.6±5.4	Contra rot: 0.6±3.2		
HSEBT	R45	Sag	**DF: 29.7±5.7**^**a**^	**Flex: 88.3±32.3**	**Flex: 108.2±7.9**^**a**^	**Flex: 51.2±6.8**	Flex: 117.7±11.5	10/12
		Front	**Ev: 12.5±4.7**	**Add: 17.2±6.5**^**a**^	Abd: 16.0±6.3	**Contra lat flex: 1.2±6.3**		
		Trans	**Abd: 11.5±4.0**	**ER: 6.1±7.9**	ER: 2.2±8.0	**Contra rot: 15.2±5.3**	ER: 36.4±18.8; Hor add: 63.7±9.2	
SEBT	R45	Sag	**DF: 32.5±5.1**^b^	**Flex: 63.9±16.1**	**Flex: 18.3±20.1**	**Flex: 8.9±13.9**		
		Front	**Ev: 2.5±3.8**	**Add: 3.2±6.2**	Add:10.3±6.7	**Contra lat flex: 0.3±13.4**		
		Trans	**Abd: 10.8±2.4**^b^	**ER: 1.5±6.2**	IR: 9.1±8.9	**Contra rot: 0.2±5.5**		
HSEBT	R90	Sag	**DF: 8.6±7.5**	**Flex: 6.6±13.6**	Ext: 0.5±11.2	Ext: 14.0±10.8	Flex: 127.9±14.6	9/12
		Front	**Ev: 18.2±3.3**^**a**^	Add: 2.7±3.0	**Add: 27.6±6.4**^**a**^	**Contra lat flex: 38.8±5.8**^**a**^	Abd:127.9±13.8*	
		Trans	**Abd: 2.1±3.4**	**IR: 1.6±3.8**	**IR: 2.1±6.0**	**Contra rot: 9.3±5.8**		
SEBT	R90	Sag	**DF: 30.2±5.5**	**Flex: 77.1±12.6**^b^	Flex: 65.2±14.0	Flex: 10.9±13.1		
		Front	**Ev: 1.5±4.1**	Abd: 1.5±6.5	**Add: 0.8±7.1**	**Contra lat flex: 0.9±12.9**		
		Trans	**Abd: 9.0±2.8**	**IR: 3.1±7.7**	**IR: 18.4±4.8**	**Contra rot: 1.1±5.4**		
HSEBT	R135	Sag	**DF: 19.7±5.7**	**Flex: 18.0±10.6**	Ext: 17.4±5.2	Ext: 28.5±9.7	Flex: 149.8±14.4	7/12
		Front	**Ev: 5.2±4.7**	Abd: 1.7±2.2	**Add: 12.1±5.2**	**Contra lat flex: 20.6±8.0**		
		Trans	Add: 0.6±4.5	I**R: 8.0±3.8**	**IR: 10.4±6.0**	Ipsi rot: 2.3±8.0	ER: 49.2±23.5	
SEBT	R135	Sag	**DF: 25.0±6.5**	**Flex: 70.4±14.4**	Flex: 84.3±10.3	Flex: 17.0±13.7^b^		
		Front	**Ev: 3.7±3.4**	Add: 3.4±7.0	**Add: 9.4±7.1**	**Contra lat flex: 0.5±16.2**		
		Trans	Abd: 4.6±4.3	**IR: 4.1±6.0**	**IR: 10.1±6.1**	Contra rot: 0.9±5.7		
HSEBT	P180	Sag	**DF: 24.5±6.4**	**Flex: 21.1±10.2**	Ext: 28.3±5.6	Ext: 36.2±7.2^**a**^	Flex: 144.3±13.0	8/12
		Front	**Ev: 0.8±2.6**	Abd: 1.6±2.4	**Add: 2.9±3.8**	**Contra lat flex: 3.2±3.6**		
		Trans	**Abd: 4.7±2.4**	IR: 2.8±3.4	**ER: 3.7±4.0**	**Contra rot: 1.8±2.8**		
SEBT	P180	Sag	**DF: 27.4±5.1**	**Flex: 75.2±10.7**	Flex: 93.8±8.8^**a**^	Flex: 18.1±13.8		
		Front	**Ev: 5.3±2.6**	Add: 11.0±6.8^b^	**Add: 13.6±4.2**	**Contra lat flex: 0.6±15.8**		
		Trans	**Abd: 7.9±2.4**	ER: 1.1±6.8	**ER: 4.4±6.9**	**Contra rot: 1.8±3.7**		
HSEBT	L135	Sag	**DF: 23.0±8.0**	**Flex: 28.8±14.0**	Ext: 29.5±6.8^**a**^	Ext: 33.9±9.7	Flex: 150.6±15.8^**a**^	6/12
		Front	Inv: 5.3±4.4	Abd: 1.8±3.4	Abd: 10.4±6.0	Ipsi lat flex: 18.3±7.9		
		Trans	**Abd: 10.2±3.0**	**ER: 5.2±5.1**	**ER: 20.4±5.5**	**Contra rot: 2.7±8.9**	ER: 50.3±25.5^**a**^	
SEBT	L135	Sag	**DF: 25.4±5.5**	**Flex: 58.0±13.4**	Flex: 78.9±14.6	Flex: 10.8±11.5		
		Front	Ev: 7.4±3.5	Add: 15.8±7.4	Add: 12.1±4.7	Contra lat flex: 0.6±18.0		
		Trans	**Abd: 7.9±2.7**	**ER: 7.8±4.9**	**ER: 17.0±7.5**	**Contra rot: 0.7±4.2**		
HSEBT	L90	Sag	DF: 9.1±9.3	Flex: 21.6±24.5	**Flex: 8.3±23.8**	**Ext: 14.8±12.9**	Flex: 130.6±12.6	4/12
		Front	Inv: 7.5±4.5^**a**^	Abd: 2.1±3.7	Abd: 16.9±6.3^**a**^	Ipsi lat flex: 38.2±7.0^a^	Abd: 129.5±13.8^**a**^	
		Trans	**Abd: 0.0±3.4**	IR: 0.1±4.9	**IR: 4.3±13.5**	Ipsi rot: 11.2±9.0		
SEBT	L90	Sag	PF: 2.3±3.4^b^	Ext: 8.7±4.8^b^	**Flex: 12.9±10.1**	**Ext: 5.1±8.7**^b^		
		Front	Ev: 12.2±3.8^b^	Add: 2.3±1.3	Add: 23.3±7.4^b^	Contra lat flex: 0.5±9.2		
		Trans	**Abd: 0.1±3.6**	ER: 6.0±5.2	**IR: 2.0±5.8**	Contra rot: 1.3±5.2		
HSEBT	L45	Sag	**DF: 19.4±8.2**	**Flex: 81.6±20.6**	**Flex: 98.8±8.2**	**Flex: 57.4±10.2**	Flex: 107.6±11.4	10/12
		Front	Inv: 1.1±5.0	**Add: 6.2±6.9**	**Add: 15.2±5.5**	Ipsi lat flex: 11.0±6.7		
		Trans	**Add: 8.2±4.9**	**IR: 12.4±6.7**	**IR: 2.1±6.0**	**Ipsi rot: 15.3±4.4**	ER: 30.4±12.7; Hor Add: 76.2±14.7	
SEBT	L45	Sag	**DF: 18.6±7.6**	**Flex: 39.7±17.7**	**Flex: 14.8±13.9**	**Flex: 3.1±10.7**		
		Front	Ev: 4.3±4.1	**Add: 2.4±6.1**	**Add: 18.4±4.5**	Contra lat flex: 4.2±9.8		
		Trans	**Add: 5.3±3.3**	**IR: 9.7±3.4**	**IR: 12.5±5.0**	**Ipsi rot: 1.3±5.6**		
HSEBT	LROT	Sag	DF: 0.7±5.2^**a**^	**Flex: 12.8±7.5**	**Flex: 10.8±5.8**	**Ext: 6.8±7.9**		10/12
		Front	Inv: 5.9±5.0	**Abd: 5.5±1.9**^**a**^	**Add: 9.8±3.7**	**Ipsi lat flex: 7.4±5.6**		
		Trans	**Add: 15.1±5.2**^**a**^	**IR: 15.1±3.7**^**a**^	**IR: 26.9±5.1**^**a**^	**Ipsi rot: 33.2±3.8**^**a**^	Hor Add: 132.8±10.7^**a**^	
SEBT	LROT	Sag	PF: 0.1±5.4	**Flex: 7.2±11.1**	**Flex: 9.2±8.4**^b^	**Ext: 3.6±7.0**		
		Front	Ev: 0.9±5.7^b^	**Abd: 2.6±2.4**^b^	**Add: 12.9±6.1**	**Ipsi lat flex: 5.9±7.2**^b^		
		Trans	**Add: 10.6±4.7**^b^	**IR: 13.7±4.8**^b^	**IR: 19.2±5.4**^b^	**Ipsi rot: 7.6±6.9**^b^		
HSEBT	RROT	Sag	**DF: 10.0±5.5**	**Flex: 6.7±11.7**^**a**^	Ext: 2.6±6.0	Ext: 2.8±8.2		8/12
		Front	**Ev: 5.9±3.4**	**Add: 3.8±2.6**	Add: 0.7±5.1	Contra lat flex: 7.2±5.5		
		Trans	**Abd: 13.4±3.6**^**a**^	**ER: 23.8±5.4**^**a**^	**ER: 29.5±5.4**^**a**^	**Contra rot: 33.7±4.5**^**a**^	Hor Add: 134.2±13.9^**a**^	
SEBT	RROT	Sag	**DF: 14.9±6.7**	**Flex: 23.4±13.9**	Flex: 12.4±8.0	Flex: 2.4±7.3		
		Front	**Ev: 4.2±4.4**	**Add: 6.2±3.9**	Abd: 5.9±7.7^b^	Ipsi lat flex: 2.0±6.3		
		Trans	**Abd: 9.1±2.9**	**ER: 16.5±5.2**^b^	**ER: 27.2±7.1**^b^	**Contra rot: 3.2±9.6**		

Shaded and white rows identify direction specific HSEBT and SEBT reach comparisons with bold font showing common joint movements

Sag, Sagittal plane; Front, Frontal plane; Trans, Transverse plane; DF, Dorsiflexion; PF, Plantarflexion; Ev, Eversion; Inv, Inversion; Abd, Abduction; Add, Adduction; Flex, Flexion; Ext, Extension; ER, External rotation; IR, Internal rotation; Ipsi, Ipsilateral; Contra, Contralateral; Lat flex, Lateral flexion; Rot, Rotation; Hor add, Horizontal adduction

^a^ = maximum magnitude of specific joint movement elicited by HSEBT

^b^ = maximum magnitude of specific joint movement elicited by SEBT

Shared joint movement synergies ranged from weak to strong (4 to 10 out of 12). Anterior and posterior reaches induced shared movement synergies of 8-10/12 and 6-8/12, respectively. Whereas, lateral and rotational reaches demonstrated shared movement synergies of 4-9/12 and 8-10/12, respectively.

The identified θ_maxHSEBT_ exhibited greater values than θ_maxSEBT_ for all joint movements, except for ankle dorsiflexion, plantarflexion and knee extension (Table 3). Joint movements with greater θ_maxHSEBT_ values were significantly greater than θ_maxSEBT_ for all comparisons, except for hip external rotation (t(34) = -0.51, p = .61, *d* = .09), with effect sizes ranging from medium to large (*d* = .39–5.21). The greater θ_maxSEBT_ values were significant for all comparisons with effect sizes ranging from medium to large (*d* = .45–1.39). Comparisons of θ_maxHSEBT_ and θ_maxSEBT_ to normative ROM values revealed that 8/22 and 3/18 joint movements, respectively, were within normative ROM values.

**Table 3:** Maximum joint movements elicited by HSEBT and SEBT with comparisons to normative ROM.

Joint	Plane	Joint Movement	Reach	θ_maxHSEBT_ (º)	Reach	θ_maxSEBT_ (º)	t-test	Cohen´s *d*	Normative ROM	HSEBT comparison	SEBT comparison
Ankle	Sag	DF	R45	29.7±5.7	R45	32.5±5.1	t(38) = 5.95 p < .01	.95	26.1±6.5^e^	x	x
		PF	LROT	-0.7±5.2	L90	2.3±3.4	t(39) = 2.91, p < .01	.45	40.5±8.1^e^		
	Front	Ev	R90	18.2±3.3	L90	12.2±3.8	t(39) = -9.46, p < .01	1.50	21±5^e^	x	x
		Inv	L90	7.5±4.5	LROT	-0.9±5.7	t(38) = -8.00, p < .01	1.28	37±4.5^e^		
	Trans	Abd	RROT	13.4±3.6	R45	10.8±2.4	t(38) = -5.45, p < .01	.87	NR	NA	NA
		Add	LROT	15.1±5.2	LROT	10.6±4.7	t(38) = -5.57, p < .01	.89	NR	NA	NA
Knee	Sag	Flex	A0	101.7±7	R90	77.1±12.6	t(39) = -9.08, p < .01	1.44	141±5.3^e^		
		Ext	RROT	-6.7±11.7	L90	8.7±4.8	t(38) = 8.67, p < .01	1.39	2±3^e^	x	x
	Front	Abd	LROT	5.5±1.9	LROT	2.6±2.4	t(38) = -7.79, p < .01	1.25	NR	NA	NA
		Add	R45	17.2±6.5	P180	11.0±6.8	t(39) = 9.04, p < .01	1.43	NR	NA	NA
	Trans	IR	LROT	15.1±3.7	LROT	13.7±4.8	t(38) = 2.45, p = .019	0.39	20^f^		
		ER	RROT	23.8±5.4	RROT	16.5±5.2	t(37) = -9.73, p < .01	1.58	30^f^		
Hip	Sag	Flex	R45	108.2±7.9	P180	93.8±8.8	t(39) = -13.37, p < .01	2.11	121±6.4^e^	x	
		Ext	L135	29.5±6.8	LROT	-9.2±8.4	t(36) = 25.92, p < .01	4.26	12±5.4^e^	x	
	Front	Abd	L90	16.9±6.3	RROT	5.9±7.7	t(37) = 7.59, p < .01	1.23	41±6^e^		
		Add	R90	27.6±6.4	L90	23.3±7.4	t(38) = 2.95, p < .01	0.47	27±3.6^e^	x	
	Trans	IR	LROT	26.9±5.1	LROT	19.2±5.4	t(37) = 10.91, p < .01	1.77	44±4.3^e^		
		ER	RROT	29.5±5.4	RROT	27.2±7.1	t(34) = -0.51, p = .61	.09	44±4.8^e^		
Trunk	Sag	Flex	A0	58.8±9.7	R135	17.0±13.7	t(38) = -18.53, p < .01	2.97	60^f^		
		Ext	P180	36.2±7.2	L90	5.1±8.7	t(38) = -18.03, p < .01	2.88	45^f^		
	Front	Lat flex	L90 and R90	38.4±6.4^a^	LROT	5.9±7.2	t(38) = -29.43, p < .01	5.21	35^f^	x	
	Trans	Rot	LROT and RROT	33.4±4.2^b^	LROT	7.6±6.9	t(38) = -21.32, p < .01	3.41	38^f^		
Shoulder	Sag	Flex	L135	150.6±15.8	NA	NA			167±4.7^e^		
	Front	Abd	L90 and R90	128.7±12.8^c^	NA	NA			184±7^e^		
	Trans	ER	L135	50.3±25.5	NA	NA			104±8.5^e^		
		Hor Add	LROT and RROT	133.5±12.3^d^	NA	NA			130^f^	x	

Shaded and white rows identify joints and regions

Sag, Sagittal plane; Front, Frontal plane; Trans, Transverse plane; DF, Dorsiflexion; PF, Plantarflexion; Ev, Eversion; Inv, Inversion; Abd, Abduction; Add, Adduction; Flex, Flexion; Ext, Extension; ER, External rotation; IR, Internal rotation; Ipsi, Ipsilateral; Contra, Contralateral; Lat flex, Lateral flexion; Rot, Rotation; Hor add, Horizontal adduction; L, Left; R, Right; A0, Anterior reach; R45, Right anterolateral (45°) reach; R90, Right lateral (90°) reach; R135, Right posterolateral (135°) reach; P180, Posterior (180°) reach; L135, Left posterolateral (135°) reach; L90, Left lateral (L90) reach; L45, Left anterolateral (45°) reach; RROT, Right rotational reach; LROT, Left rotational reach; NA, Not applicable; NR, Not reported; x, within normative ROM.

^a^ Average trunk lateral flexion L90 and R90 reach

^b^ Average trunk rotation LROT and RROT reach

^c^ Average shoulder abduction L90 and R90 reach

^d^ Average shoulder horizontal adduction LROT and RROT reach

^e^ Reference value from Greene and Heckman [25]

^f^ Reference value from Magee [26]

## Discussion

The current study established that the HSEBT provides additional information about dynamic postural control and functional mobility. However, there seems to exist a relationship since total scores (A_tot_ and CS_tot_) have demonstrated fair to moderate correlations. Nevertheless, large reach specific differences were noted. Anterior HSEBT reaches are closer related to both anterior and posterior SEBT reaches, which can be partially explained by stronger shared movement synergies. Posterior and lateral HSEBT reaches demonstrated weaker relationships to their SEBT counterparts, indicating that these tests measure different aspects of dynamic postural control. Overall, the HSEBT elicited greater joint movements (θ_maxHSEBT_) than the SEBT (θ_maxSEBT_). In addition, 8/22 θ_maxHSEBT_ were within normative ROM values, while θ_maxSEBT_ had only 3/18 joint movements within normative ROM values. These findings may justify the application of the HSEBT as a useful clinical tool in the assessment of functional mobility.

### Dynamic postural control

HSEBT is able to measure different aspects of dynamic postural control in comparison to the SEBT. The strength of the shared movement synergies could explain some of the differences observed. The lateral reach with a weak movement synergy (4/12) had little to no correlations, while the lateral reach with a strong movement synergy (9/12) had fair to moderate correlations. Furthermore, posterior reaches had moderate shared movement synergies (6-8/12) and fair correlations, while rotational and anterior reaches with moderate to strong shared movement synergies (8-10/12) had fair to good correlations (Table 2, Figs 2 and 3). Since anterior HSEBT (A0, R45 and L45) and posterior SEBT (P180, L135 and R135) reaches also had strong shared movement synergies (8-11/12, obtained from Table 2) and joint movements of a more similar magnitude, especially hip joint (Table 2), an anterior HSEBT to posterior SEBT CS comparison should not influence the moderate to good anterior CS correlations. However, correlation coefficients all decreased for these comparisons (Table 1). Thus, it appears that a shared movement synergy is only one of the plausible explanations for the variable correlations between the reaches. Specific joint movements of a shared movement synergy, as observed in the ankle, may have a greater influence considering that dorsiflexion was found to predict anterior SEBT reach performance [14, 30]. However, the influence of dorsiflexion on anterior HSEBT reach performance has not been established. Another reason for the differences in the correlations between reaches may lie in the similarity of balance boundary conditions. This could explain why the anterior HSEBT and SEBT CS comparisons had stronger correlations than the anterior HSEBT and posterior SEBT CS comparisons. Future studies utilizing center of pressure analysis should investigate this hypothesis. In addition, the influence of vision could also have influenced the anterior and posterior comparisons, since visual feedback of the reaching target was available for anterior, but not for posterior reaches. Composite scores of right foot anterior reaches and the R45 reach had good correlations, while the remaining comparisons yielded none to moderate correlations. This suggests that the HSEBT is able to measure some different aspects of dynamic postural control as compared to the SEBT.

### Functional mobility

The multi-joint movements observed for the different maximum hand reaches are organized to meet the task requirements and to overcome internal constraints. These internal constraints include not only postural and balance control strategies, but also individual joint movement capacities. Thus, reach measurements provide information of how the body is able to organize and utilize joint excursions in a more ecological way. The HSEBT is therefore an appropriate measure of functional mobility since it is the result of joint movement combinations of the lower extremity, trunk and shoulder.

The data presented here provide not only a reference for functional mobility (Figs 2 and 3), but also reference values of joint movements (θ) and their combinations elicited for all HSEBT reaches in a young and healthy male population (Table 2). Our kinematic data, as well as data from other studies [10–14], demonstrated that hand reaches resulted in more joint movements than foot reaches alone. Furthermore, θ_maxHSEBT_ were significantly greater for trunk, hip (except external rotation), knee, ankle, and upper extremity than θ_maxSEBT_ (Tables 2 and 3). In addition, θ_maxHSEBT_ were also more consistent within normative ROM values (8/22 joint movements) in comparison θ_maxSEBT_ (3/18 joint movements). The greater joint movements observed with the HSEBT (θ_maxHSEBT_) might be due to the larger base of support in the HSEBT, whereby decreasing the balance and postural control demand. Thus, the HSEBT appears to be a good alternative to quantify functional mobility.

The HSEBT quantifies functional mobility in the sagittal (A0 and P180), frontal (L90 and R90) and transverse planes (LROT and RROT). The plane specific capacity of these reaches is reflected by its ability to elicit one or more θ_maxHSEBT_ in their respective planes of motion. (Table 3). Since decreased ROM of specific joints have been found to impact joint movements elsewhere in the kinetic chain [31, 32], the HSEBT could be used to assess the influence of joint mobility limitations on functional mobility. One approach could be to measure multiple hand reaches to explore if specific joint mobility limitations could be identified. For example, anterior reaches (L45, A0 and R45) resulted in both common and different joint movements (Table 2). These flexion movement patterns, based on common ankle dorsiflexion, knee and hip flexion, elicit different frontal and transverse plane movements. The decrease in anterior reach values in L foot R45 compared to L45 suggests that sagittal plane joint movements of the lower extremity are influenced by frontal and transverse plane joint movements. More specifically, less dorsiflexion was observed with inversion and adduction (L45) than with eversion and abduction (R45) (Table 2). These findings are supported by the work of Tiberio and co-workers who showed that a pronated ankle yielded greater dorsiflexion [33]. Furthermore, L45 hand reach resulted in less hip flexion when compared to R45. This could be explained by the impact of both hip internal rotation and adduction (in the L45 reach) approaching positions of bony impingement as previously described in the literature [34]. In contrast, the hip external rotation and abduction associated of the R45 reach did not approach positions of bony impingement [34]. Thus, both L foot L45 and R foot R45 hand reaches can be used as a weight bearing version of a common clinical test for femoroacetabular impingement (FAI), which is currently done in supine with hip passively brought into flexion, adduction and internal rotation (FADIR).

Frontal and transverse plane trunk movements are opposite for the L45 and R45 reaches possibly having an influence on the reach results. However, these opposite movements are less than 50% of observed θ_maxHSEBT_ (Tables 2 and 3) suggesting that these trunk movements do not impact reach measurements significantly. Similar to the anterior reach analysis, posterior reaches or extension movement patterns based on a common hip extension, can be analyzed to determine the influence of frontal and transverse plane joint movements on extension.

The HSEBT and SEBT elicited 8 of 22 and 3 of 18 joint movements that were within normative ROM values, respectively. This is not surprising considering that joint ROM measurements are usually obtained using goniometry in positions that do not require neither strength nor neuromuscular control. Furthermore, the transfer of joint ROM to functional tasks has only limited significance [35]. Considering that the HSEBT elicited more and greater trunk, upper and lower extremity joint movements coupled into one functional unit [36], the HSEBT may also be a good assessment tool for functional mobility.

### Clinical application

The HSEBT has the potential to have complementary and wider clinical application possibilities than the SEBT, which is primarily used in the assessment of the lower extremity function [27, 37–43]. Since the HSEBT integrates more and greater joint movements of the full kinetic chain, it might find clinical applications in e.g. low back pain (LBP), where the assessment of full-body movements has been reported as underexplored [21]. Furthermore, in patients with shoulder dysfunctions hand reaches can provide important clinical information since dynamic positioning of the scapula to stabilize the glenohumeral joint is dependent on the segmental coordination of the entire kinematic chain [20]. In addition, the HSEBT could be useful in fall risk management since falling occurs while reaching, leaning [44] and bending [45]. Currently, a single item hand reach test, the functional reach test [16], and the multi-directional reach test [18] are used to quantify limits of stability in populations at risk. However, these tests only include reaches at shoulder level, neither provoking overhead activities nor bending. Thus, the HSEBT might be an alternative tool in fall risk management. Furthermore, the HSEBT can be useful in the assessment of athletes participating in overhead sports such as throwing (baseball and European handball) and hitting (tennis and golf).

## Conclusions

In comparison to the SEBT, the HSEBT measures different aspects of dynamic postural control, especially in the posterior and lateral reaches. Shared movement synergies could explain some of the observed relationships between both tests. Considering that the HSEBT elicit more and greater joint movements than the SEBT, and that there is no currently available functional mobility assessment tool, the HSEBT may also present a useful addition to the available test methods of functional mobility.
